# Paternal Fenitrothion Exposures in Rats Causes Sperm DNA Fragmentation in F0 and Histomorphometric Changes in Selected Organs of F1 Generation

**DOI:** 10.3390/toxics9070159

**Published:** 2021-07-05

**Authors:** Nur Afizah Yusoff, Izatus Shima Taib, Siti Balkis Budin, Mahaneem Mohamed

**Affiliations:** 1 Biomedical Science Programme, Centre of Diagnostic, Therapeutic and Investigative Studies, Faculty of Health Sciences, Universiti Kebangsaan Malaysia, Jalan Raja Muda Abdul Aziz, Kuala Lumpur 50300, Malaysia; afizah.yusoff@yahoo.com (N.A.Y.); balkis@ukm.edu.my (S.B.B.); 2 Department of Physiology, School of Medical Sciences, Universiti Sains Malaysia, Kubang Kerian 16150, Malaysia; mahaneem@usm.my

**Keywords:** histomorphometry, infertility, organophosphate, progeny, reproductive toxicity, sperm DNA damage

## Abstract

The adverse effects of maternal pesticides exposure on the progeny is very well established. However, the impact of paternal exposure to pesticides such as Fenitrothion (FNT) on the histomorphometry of progeny’s organs in unexposed mothers are much less well studied. Therefore, this study aims to evaluate the effects of paternal FNT exposure on the sperm quality of the parent rat and its effects on the histomorphometry of the progeny’s organs. Randomly, male Sprague Dawley rats (*n* = 24) categorized as F0 were distributed equally into three groups namely Control, FNT-10, and FNT-20. Control received 1 mL/kg corn oil while FNT-10 and FNT-20 received 10 mg/kg and 20 mg/kg of FNT, respectively, via oral force feeding for 28 consecutive days. At the end of the study, male rats were mated with unexposed female rats and the male rats were sacrificed to obtain sperm for sperm characterization and DNA damage evaluation. Meanwhile, the rats’ progeny (F1) namely *p*Control, *p*FNT-10, and *p*FNT-20 were left to grow until postnatal day 70 before being sacrificed to obtain the matured organs for histology and morphometric analysis. Our results showed that both doses of FNT reduced sperm quality and caused DNA fragmentation in F0 rats compared with the control group (*p* < 0.05). The number of Leydig cells as well as the diameter of the seminiferous tubules and glomerulus of the *p*FNT-20 group had significantly decreased (*p* < 0.05) compared with the *p*Control group. The Bowman’s space of the *p*FNT-20 group had significantly increased (*p* < 0.05) compared with the *p*FNT-10 and *p*Control groups. Therefore, paternal exposure to FNT reduced the sperm quality and increased sperm DNA fragmentation in F0 male Sprague Dawley rats and altered the histology and morphometry of the selected organs in the F1 progeny.

## 1. Introduction

Environmental toxicants including organophosphate (OP) have been shown to cause harmful effects particularly on animal reproduction and sperm. Human sperm can develop chromosome aneuploidy, chromatin alterations, increased sperm DNA damage, and lower sperm concentrations as a result of OP pesticides poisoning [1,2]. This OP poisoning can occur through inhalation, dermal, and ingestion, the three main routes of entry into the human body [3]. Similar to other OP pesticides, Fenitrothion (FNT) (O,O-dimethyl-O-(3-methyl-4-nitrophenyl) phosphorothioate) also exerts its adverse effect by the accumulation of neurotransmitter acetylcholine (ACh) due to the inhibition of acetylcholinesterase (AChE), which leads to severe consequences such as seizures, respiratory failure, and eventually death [4]. FNT is a broad-spectrum OP pesticide that is commonly used as a vector control agent in the public health sector besides its usage in controlling pests such as mites and insects in the agricultural sector [5]. Therefore, due to its extended use, FNT is found persistently in the environment [6]. Humans are potentially exposed to FNT, particularly in the soil at a concentration of more than 2 mg/kg in the environment [7]. Furthermore, humans may also be exposed to FNT either indirectly via food consumption or directly through occupational exposure [8]. FNT has been shown to cause detrimental effects on the liver [9], lungs [10], and kidney [11] of rats. It is also reported to induce oxidative damage in many organs such as testis and sperm [12].

Reproductive toxicity is generally manifested by alterations in the onset of puberty, sexual behavior and performance, premature reproductive senescence, production and transportation of gametes, and infertility and loss of the fetus during pregnancy, all of which are reliant on the reproductive system’s integrity in both females and males [13]. FNT has been reported to alter the reproductive performance and sexual behavior in male Sprague Dawley rats [14]. Infertility is identified as an alarmingly worldwide problem with a predictable 48.5 million couples being infertile in 2010 alone [15]. Infertility is defined as a disease characterized by the failure to establish a clinical pregnancy after 12 months of regular, unprotected sexual intercourse or by a reduction in a person’s ability to reproduce, either alone or with a partner [16]. Men have contributed to about 50% of the causes of infertility [15]. Anatomical abnormalities such as varicocele [17], oxidative stress, genetic defects, hormonal imbalance, and inappropriate diet are among the factors that contribute to male infertility [18]. Moreover, toxic agents such as pesticides, radiation, and drug exposure also play an important role in contributing to infertility [19]. Several studies reported that antiandrogenic effects [20,21] and oxidative sperm DNA damage [22] have been linked as the male reproductive system defect-causing mechanisms for FNT and its metabolite. A previous study showed that malformed or aborted children are associated with reactive oxygen species (ROS) levels and DNA fragmentation in the semen of male workers exposed to radiation [23]. Apoptosis, impairment of sperm chromatin maturation, and oxidative stress are among the mechanisms involved in inducing sperm DNA fragmentation. Sperm cell has been identified as a vector in paternal toxicant exposure because it will carry the DNA damage-induced by the toxicants [23,24]. This DNA damage also known as epigenetic marks can be passed to the progeny through the semen upon fertilization with the ovum. Most, but not all, DNA damage carried by the sperm can be reprogrammed after fertilization. Therefore, the persisting DNA damage can lead to the abnormal genetic expression in the progeny [24].

Puberty or sexual maturation is the end point for a complex sequence of early development and progression in gaining reproductive competency. Internal and external genitalia in response to hormonal signals from the hypothalamic-pituitary gonadal (HPG) axis somehow need to be matured, hence successfully allowing fertilization [25]. Moreover, the transmissible effects of environmental toxicants such as FNT, including genomic instability, sperm DNA mutations, imprinting errors, and apoptosis have been proposed to be affected by epigenetic modifications [26]. It is characterized by histone modifications, chromatin remodeling, and DNA methylation that are important regulators in the spermatogenesis during sperm maturation [27] and proper embryonic development [25,28]. FNT metabolite known as fenitrooxon has been reported to be involved in hepatic lipid [9] and sperm DNA strand breaks in rats [29], hence altering fertilization and the developing fetus. Growing evidence in animal models suggests that immediate adverse effects involving methylation and gene transcription as well as long-term pathologies in the embryo, fetus, and the offspring such as tumors have occurred in the fertilization of DNA-fragmented spermatozoa [28].

Paternal exposure to di-N-butyl-phthalate, which is a type of phthalate has been reported to deteriorate the development of female progeny and characterized by delays in sexual maturation as well as reduction in the sperm quality of male rat’s progeny [30]. However, exposure to FNT at the dose 10, 20, and 60 ppm in the diet for 10 weeks in utero and from weaning to maturation did not cause any defects in progeny [31]. Moreover, to the best of our knowledge, the effect of paternal exposure of FNT towards the developmental landmarks and organs of first-generation progeny from an unexposed mother has never been reported. In order to monitor the antiandrogenic effect of FNT, developmental landmarks such as nipple retention and anogenital distance (AGD) can be used since they have been associated with androgen-dependent reproductive tissues. In addition, the effects of sperm DNA fragmentation found during paternal exposure of FNT in influencing organ development of the progeny is under-reported. Sperm DNA fragmentation may influence the development of the progeny [32]. Hence, this study aims to assess the male-mediated reproductive toxicity effects of different doses of FNT on first-generation progeny (F1) of rats via developmental landmarks as well as the histology and morphometry of the organs. The doses used in the current study are postulated to be 1 mg/kg and 2 mg/kg of the human dose, which is based on the theory that when comparing with laboratory animals such as rats, humans are more sensitive at 10 times to OP [7]. Even though the doses used in the current study were higher compared with the acceptable daily intake (ADI) for FNT, the type of exposure is a short-term acute exposure, which requires a higher concentration of chemicals to be used in the toxicity evaluation [33]. Furthermore, based on the fetotoxicity findings, Turner and colleagues [34] concluded that the lowest observed adverse effect level (LOAEL) for the developmental toxicity of progeny in rat was reported to have decreased from 25 mg/kg to 20 mg/kg. Therefore, this study might provide new information on the developmental landmarks as well as the histology and morphometry of the organs focusing on F1 progeny when only paternal male rats (F0) are exposed to FNT in a short-term acute exposure toxicity study.

## 2. Materials and Methods

### 2.1. Chemicals

FNT with purity of 98.66% was obtained from LGC Labor GmbH (Augsburg, Germany; Lot No. G144531; CAS Number: 122-14-5). FNT at the dose of 10 mg/kg and 20 mg/kg were prepared by diluting with corn oil. Other reagents and chemicals were bought from Sigma-Aldrich (St. Louis, MI, USA) with high purity grade. 

### 2.2. Experimental Animals

A number of 24 fertile male Sprague Dawley rats weighing 240–270 g (9 weeks old) were provided by the Laboratory Animal Research Unit of the Health Campus in Universiti Sains Malaysia (USM), Malaysia. Polycarbonate cages (BPA free) were used to house all of the rats with 2 animals per cage in a control environment (20–24 °C, reversed 12 h light/dark cycle, and relative humidity of 50 ± 5%). The rats were given one week for acclimatization prior to experimentation. The rats were given rodent chow pellets (Gold Coin Sdn. Bhd. Kuala Lumpur, Malaysia) and water ad libitum. All animals were strictly handled in accordance with the ethical guidelines approved by the Animal Ethics Committee of Universiti Sains Malaysia with reference no. USM/IACUC/2018/(112)(921) and the Universiti Kebangsaan Malaysia Animal Ethics Committee (UKMAEC) with reference no. FSK/2016/IZATUS/23-NOV./807-NOV.-2016-FEB.-2019.

### 2.3. Experimental Design

Randomly, all male rats (F0) were further divided into three groups with 8 rats per group. The control group (C) received corn oil at the dose of 1 mL/kg; whereas FNT-10 received 10 mg/kg/day FNT (1/60 LD50) [35] and FNT-20 received 20 mg/kg/day FNT (1/30 LD50) [12]. All of the substances were administered via oral forced feeding using needle gavage for 28 consecutive days between 09:00 a.m. and 10:00 a.m. [12]. After 4 weeks of treatment, two proven fertile female rats in the oestrous phase were paired with each male rat. However, before pairing with the male rats, the female rats were first screened for two consecutively regular oestrous cycles. They were paired during the dark phase of the reversed light/dark cycle between 9:00–12:00 h for 3 h per day [36]. After the mating period, by using a light microscope (Olympus BX41, Olympus Corporation, Tokyo, Japan), vaginal smears were observed for the presence of spermatozoa. The day was recorded as day 0 of pregnancy (confirmed mating) when there was spermatozoon positive in the vaginal smear [37]. Female rats were left until giving birth to progeny, starting from day 21. After mating, the male rats were anaesthetized with a single intraperitoneal injection of ketamine and xylazine cocktail (KTX) prior to being sacrificed [14]. For evaluating the sperm characteristics and DNA fragmentation, the sperm were collected from the cauda epididymis of F0 male rats. Meanwhile, the rat’s progeny (F1), namely *p*Control, *p*FNT-10, and *p*FNT-20, were left to grow until postnatal day 70 for evaluation of developmental landmarks. At the end of the study, the selected organs from both sexes of F1 rats were used for histomorphometric analysis.

### 2.4. Sperm Characteristics Analysis

After dissection, the sperm was collected immediately and was suspended in Hank’s balanced salt solution (HBSS) with 298 mOsmol/kg, pH 7.4. For the epididymal sperm count and motility analysis, a total of 10 μL of sperm suspension was placed on a Makler counting chamber (Sefi-Medical Instruments, New York, NY, USA). The sperm motility was expressed in percentage of motile sperm while sperm count was expressed as million sperm cells per ml of suspension. Meanwhile, for sperm viability assessment, a thick smear was done using 10 μL of sperm suspension and adding 10 μL of eosin-nigrosin stain on the slides. The dead sperm will take up the eosin stain and appear pinkish while normal live sperm will not take up the eosin stain and appear white in color. In order to assist the observation, a thin smear of sperm suspension using a Diff-Quik staining kit was done and the percentage of abnormal sperm morphology was calculated. The morphological abnormalities of 200 sperms were examined per slide under oil immersion. The data are obtainable as a percentage of abnormal sperm morphology. The sperm characteristics analysis was performed in triplicate per rat in accordance with the guidelines by [38] while guidelines by [39] were used to analyze the rat sperm abnormal morphology.

### 2.5. Sperm DNA Fragmentation Analysis

Sperm smears were air-dried at room temperature for 1 h on glass slides and fixed in Carnoy’s solution (methanol/glacial acetic acid, 3:1) at 4 °C for 2 h. Thereafter, they were stained for 10 min in freshly prepared acridine orange (0.19 mg/mL in Mcllvain phosphate-citrate buffer, pH 4.0). The smears were then examined under a fluorescent microscope (Olympus BX41) with a 460 nm filter [40]. Two slides were stained for each rat and the number of spermatozoa with fragmented DNA (yellow and dark orange fluorescences) in 100 spermatozoa/field was counted accordingly.

### 2.6. Developmental Landmarks Evaluation

The developmental landmarks of anogenital distance (AGD) and number of nipple and areola were measured in both male and female F1. A digital caliper and magnifying glass were used to measure AGD on PND0 (postnatal day 0) and on PND12 and PND35; AGD was measured without a magnifying glass. The distance was measured from the genital part to the caudal of the anus. In addition, the number of nipple or areola of F1 was recorded for both female and male rats in PND12. The observations were scored based on the discoloration around the nipples and the presence or absence of nipple buds [41].

### 2.7. Histomorphometry of Progeny’s Organ Analysis

The other organs were fixed in 10% formalin, while the right testis and epididymis of each rat were fixed in Bouin’s solution overnight, then dehydrated and embedded in blocks of paraffin. Haematoxylin and eosin (H&E) were used to stain sections of 5 µm thickness and viewed under a light microscope (Olympus BX41). Epididymal epithelial thickness of the testis, epididymis, prostate gland, seminal vesicle, ductus deferens, and uterus as well as other organs were measured using the Image J software. The number of Leydig cells in 20 random intertubular areas (area enclosed by three seminiferous tubules) was counted using 40× magnification for the Leydig cell count [42]. A total of 10 randomly selected seminiferous tubules were used to determine the mean Johnsen testicular biopsy score (MJTBS) by using the method shown in Table 1, as reported earlier [42]. A round line was drawn on the cardiomyocytes in 10 randomly selected slides and measured using 40× magnification [43]. Meanwhile for the liver, a square line was drawn in 10 randomly selected areas of each group and measured using 40× magnification [44]. For renal, lines were drawn on 100 randomly selected glomerulus and Bowman’s space and measured using 40× magnification [45]. The number of alveoli was counted from the intercepting line between the alveolar walls [46]. All of these histomorphological changes were verified by a pathologist.

### 2.8. Statistical Analysis

The Statistical Package for the Social Sciences (SPSS) version 23 was used to analyze the data. The normal distribution data were further analyzed with one-way analysis of variance (ANOVA) followed by the Tukey post hoc test. The results were expressed as mean ± standard error of the mean (SEM) and the differences were statistically significant at *p* < 0.05.

## 3. Results

### 3.1. Sperm Characteristics

**Figure 1:**
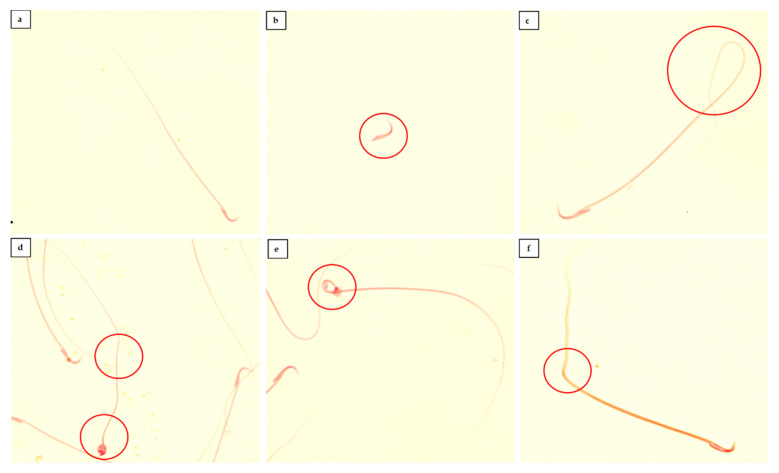
Comparison of normal and abnormal sperm morphology, 40×. (**a**) Shows normal sperm morphology; hook head and long tail. (**b**) Shows abnormal tailless sperm. (**c**) Sperm with coiled tail. (**d**,**e**) Depicts a bend at a point on the sperm tail and abnormally developed sperm head such as pin and amorphous. (**f**) Cephalocaudal bending. Sperm was stained with a Diff-Quik staining kit.

The sperm characteristics of the experimental paternal male rats are shown in Table 2. Treatment of FNT significantly lowered the epididymal sperm count, motility, and viability as well as increased the percentage of sperm with abnormal morphology in rats (*p* < 0.05). Furthermore, when compared with the FNT-10 group, sperm count, viability, and motility were significantly lower but were higher in abnormal sperm morphology in the FNT-20 group (*p* < 0.05). Figure 1 depicts the differences in normal and abnormal morphology of sperm.

### 3.2. Sperm DNA Fragmentation

**Figure 2:**
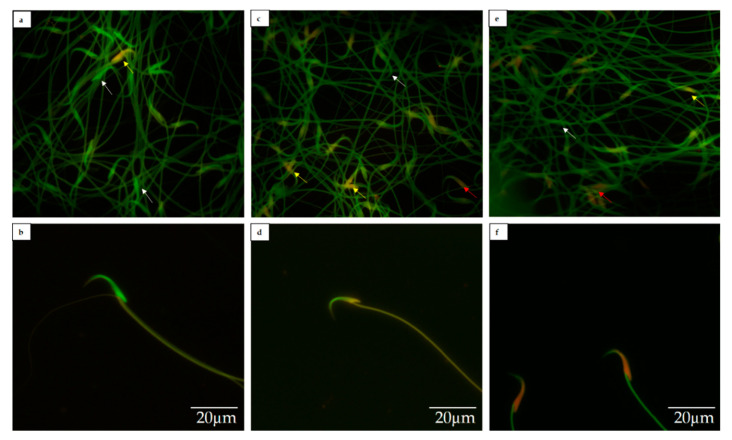
Sperm nuclear DNA fragmentation using the acridine orange test. Sperm smears were stained with freshly prepared acridine orange and viewed using a fluorescent microscope (oil immersion) and a 460 nm filter (scale bar: 20 µm). (**a**) Control, 100×; (**b**) sperm heads with green fluorescence, 100×; (**c**) FNT-10, 100×; (**d**) sperm heads with yellow fluorescence, 100×; (**e**) FNT-20, 100×; (**f**) sperm heads with dark orange fluorescence, 100×. Sperm heads with green fluorescence (white arrow) indicate intact DNA while sperm heads with yellow (yellow arrow) and dark orange fluorescence (red arrow) indicate fragmented DNA.

The sperm DNA fragmentation in all groups is shown in Table 2. The result shows that sperm DNA fragmentation was significantly higher in FNT-10 and FNT-20 groups compared with the control group (*p* < 0.05). Moreover, when compared with the FNT-10 group, sperm DNA fragmentation was significantly higher in the FNT-20 group (*p* < 0.05). This result is also illustrated in Figure 2 in which the sperm heads with green fluorescence (white arrow) indicate intact DNA while sperm heads with yellow (yellow arrow) and dark orange fluorescence (red arrow) indicate fragmented DNA.

### 3.3. Developmental Landmarks Evaluation

**Figure 3:**
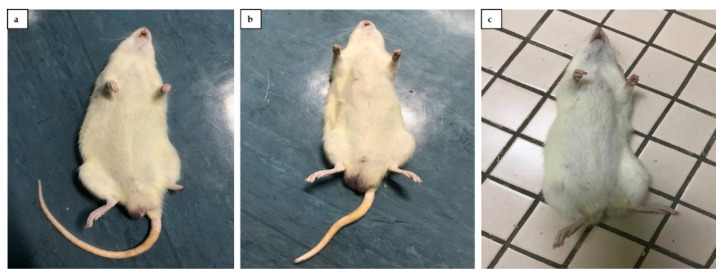
Gross anomalies observed in F1 progeny of *p*FNT-20 rats. (**a**) Defective foot. (**b**) Short tail. (**c**) No tail.

Table 3 shows the developmental landmarks of the experimental rats. No significant difference was observed (*p* > 0.05) in all parameters of all groups such as anogenital distance as well as the number of nipples and areola. However, three F1 progeny of *p*FNT-20 rats showed gross anomalies such as short or no tail as well as defective foot, however, this finding was not significant when compared with the total number of F1 progeny (Figure 3).

### 3.4. Absolute and Relative Weight of Organs

The findings on absolute and relative weight of organs of all the rats’ progenies are presented in Supplementary Table S1. The absolute weight is the actual organ weight while the relative weight is the proportion of the organ weight towards the body weight in percentage. Overall, parental FNT exposure was found to not significantly impact the absolute and relative weight of male and female F1 organs. 

### 3.5. Histomorphometry Analysis

The testicular morphometry of the experimental rat is shown in Table 4. The Leydig cell count was found to decrease in the *p*FNT-20 group compared with the *p*Control group. The seminiferous tubule diameter in the rat of the *p*FNT-20 group was significantly smaller compared with the *p*Control group (*p* < 0.05). In addition, this study also showed no significant difference in the seminiferous tubule epithelial height, seminiferous tubule with germ cell loss, and Johnsen testicular biopsy score in all experimental groups. There were no histological changes observed in male reproductive organs as well as in the ovary and uterus in female progenies as shown in Supplementary Figures S1 and S2. Furthermore, Supplementary Table S2 shows no significant difference in the epididymis, prostate gland, seminal vesicle, ductus deferens, and endometrium epithelial height as well as endometrium wall thickness of F1 progeny in all experimental groups.

**Figure 4:**
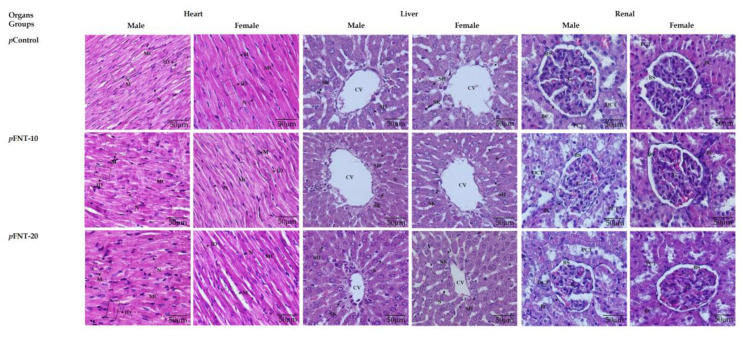
Heart, liver, and renal cross section of rats stained with H&E. (Magnification: 40×). Normal myocardiocyte (MC) is characterized by a single nucleus (N). The myofibril (M) is arranged in an orderly manner with the presence of striation on MC in both male and female groups. Myocardiocyte cells (MC) are connected between each other through an intercalated disc (ID) that is located in the end of MC. The cubical shape of the hepatocyte cell (SH) looks normal along the central vein (CV) and sinusoid (S). The Kupffer cell (SK) in star shape in S was observed in all groups. However, CV was smaller in *p*FNT-20 compared with other groups. Normal glomerulus (G), Bowman’s capsule (BC), Bowman’s space (BS), distal convoluted tubule (DCT), and proximal convoluted tubule (PCT) were observed in all female rat groups. There was atrophy in the glomerulus (G) size and Bowman’s space (BS) dilatation in male rats of *p*FNT-10 and *p*FNT-20 groups.

Table 5 shows no significant difference in the size of cardiomyocytes and hepatocytes as well as the number of alveoli in both genders of F1 progeny among all experimental groups. The structure of myocardium is also normal with even myofibril arrangement and striation in the *p*Control, *p*FNT-10, and *p*FNT-20 groups (Figure 4). Figure 4 also shows histological observation of liver F1 progeny at 40× magnification. Overall, the structure of the hepatic lobule was normal in the *p*Control, *p*FNT-10, and *p*FNT-20 groups. Hepatocyte cells were observed to be in a well-organized arrangement with a cubical shape along the central vein and sinusoid. However, a smaller central vein was observed in the *p*FNT-20 group.

Normal stellate Kupffer cells (star shape with ovoid nucleus) were located in the sinusoid layer in all three groups. Histological observations of the lung are shown in Supplementary Figure S3 at 40× magnification. All experimental groups for both male and female rats showed a normal alveolar structure and spleen with presence of an intact germinal center.

However, the glomerulus diameters in the *p*FNT-10 and *p*FNT-20 male groups were significantly lower (*p* < 0.05) compared with the *p*Control group. Meanwhile, the Bowman’s space areas in the *p*FNT-10 and *p*FNT-20 male groups were significantly higher (*p* < 0.05) compared with the *p*Control group. The Bowman’s space area in the *p*FNT-20 male group was also significantly higher (*p* < 0.05) compared with the *p*FNT-10 group. The renal morphology was normal with intact glomerulus, Bowman’s capsule, and Bowman’s space in the *p*Control, *p*FNT-10, and *p*FNT-20 groups (Figure 4). No changes were seen in the proximal convoluted tubules and distal convoluted tubules in all groups. However, there was some glomerulus size atrophy and dilatation of Bowman’s space in male rats of the *p*FNT-10 and *p*FNT-20 groups. Lastly, Supplementary Figure S3 also shows a spleen with a normal morphological structure and the presence of an intact germinal center in all experimental groups.

## 4. Discussion

### 4.1. Sperm Characteristics

Reproductive impairment, which eventually leads to infertility, is one of the most often overlooked consequences of OP exposure in males of reproductive age. Some previous studies have reported on the detrimental effects of FNT on sperm. Certainly, oxidative stress was identified as a mechanism involved in FNT-induced sperm DNA damage [12]. Sperms are highly vulnerable to oxidative damage attributable to its high polyunsaturated fatty acids (PUFAs) content and low antioxidant protection and are very susceptible to the ROS attack [47]. This reflects the finding of this study that showed both doses of FNT reduced the sperm quality by reducing the sperm motility, count, and viability, and increasing abnormal morphology. These findings are in agreement with previous studies that mentioned that inhibition of enzymatic antioxidant activity as well as increase in lipid peroxidation were found to be involved in the oxidative stress mechanism in reducing the sperm quality following OP pesticides exposure [22,48]. FNT, like other OPs, has been reported to be an antiandrogenic agent and mimics the oestrogen hormone that leads to the disruption of testosterone circulation [49]. In the end, this disturbance causes changes in spermatogenesis in the testis and decreases sperm synthesis [50]. Fatty acid amide hydrolase (FAAH) plays several vital roles in sperm motility acquisition and spermatogenesis by regulating apoptosis or mitochondrial activity [51]. However, down-regulation of FAAH by fenitrooxon will continuously stimulate the cannabinoid signal, leading to apoptosis of testicular cells such as the Sertoli and Leydig cells. This will cause an imbalance of hormone regulation such as for testosterone, which potentially led to the reduction in sperm quality in this study.

### 4.2. DNA Fragmentation

In the present study, FNT was proven to cause an increase in the sperm DNA fragmentation. Sánchez-Peña and colleagues [52] reported that about 75% of Mexican workers who had been exposed to OP showed a DNA fragmentation index (DFI) of more than 30% compared with those not exposed to OP, who only showed 9.9% of DFI. A previous research reported that male rats given artesunate, an antimalarial agent, experienced sperm DNA strand breaks as seen through a comet assay evaluation [53]. One of the causes involved in the OP-induced sperm DNA damages is oxidative stress. Spermatozoa are vulnerable to free radicals due to their membranes that are rich in PUFAs, leading to lipid peroxidation. The final result of lipid peroxidation is mutagenic and genotoxic, which eventually affects the DNA [54]. Furthermore, DNA repair is limited within the spermatozoa and only happens during specific processes of the spermiogenesis. During nuclear condensation in the epididymis, the repair mechanism is no longer activated [55,56]. Moreover, OP is considered as a potent phosphorylating agent in animals due to its ability to change the chromatin structure via protamine for DNA binding. This condition will cause the DNA to be exposed to the induction of denaturation in situ [57].

### 4.3. Developmental Landmarks

Interestingly, the damaged DNA which is carried by the sperm has the possibility to be repaired by oocytes. However, the damaged sperm has a significant effect on fertilization and its viability before reaching the oocytes. It will also reduce the fertilizing capacity and pregnancy outcomes [58,59]. Our previous study reported that parental exposure of FNT reduced the reproductive performance and pregnancy outcomes [14]. Even though parental exposure of FNT was proven to impair the reproductive performance and pregnancy outcomes, its effects towards the F1 progenies is still uncertain. Indicators of the F1 progenies’ developmental landmarks have been investigated for evaluating the antiandrogenic effect including AGD and the number of nipples or areola for both male and female progenies. Both sexes can exhibit different responses from toxicant exposures influenced by cellular and molecular processes as well as interactions between environmental chemicals and physiological molecules [60]. Furthermore, environmental toxicants present in seminal fluid have the potential to transmit the effects of paternal exposures to the offspring [61]. The current findings showed that paternal exposure to FNT did not cause any changes in the developmental landmarks among all progeny groups. These results are contradictory to a previous study, which found that bupropion hydrochloride (BUP) administration decreased AGD in both male and female rats [62]. 

No significant changes were observed in the development landmarks in the current study, probably due to the rapid FNT metabolism in the liver of the parental rats. Biotransformation of FNT by cytochrome P450 in the liver resulted in the formation of a reactive metabolite known as fenitrooxon [63]. This metabolite has been reported to not have any antiandrogenic activity [20], thus explaining the absence of antiandrogenic effects in the F1 progeny. For males, short AGD indicates disruption androgen action while for females, a long AGD indicates masculinization effects caused by a high androgen level or AR ectopic activation [64]. In this study, some anomalies such as short and absent tail as well as rats with no feet were observed in the F1 progeny of the FNT-20 group. These findings are supported by a previous study in which male preconception exposure to ethyl nitrosourea or urethane induced malformations and tumors in several generations of progeny [65]. Furthermore, FNT is suggested to be epigenetically toxic; hence, heritable changes in gene expression may occur without changes in the DNA sequence during fertilization. In addition, genome aberrations by DNA methylation during the early stage of embryo development may also influence organ development defects in the embryo [66]. 

### 4.4. Histomorphometry Analysis

The disruption of androgen hormones, especially testosterone, not only caused changes in organ weight but also altered the function, histology, and morphometry analysis of reproductive organs such as the testes, prostate gland, epididymis, seminal vesicle, ductus deferens, ovary, and uterus [67,68]. However, in the present study, all male and female reproductive organs of the F1 progeny in the FNT group were normal based on the histology and morphometric analysis when compared with the F1 progeny of the control group. These findings are in alignment with a previous study by Okahashi and colleagues [31] which showed that even when FNT was given directly to the progeny, it still did not cause any morphological changes to the organs. However, the height of seminiferous epithelium and the number of Leydig cells found in the testes of both F1 progeny groups of FNT were significantly decreased compared with the control group. Wilson and colleagues [69] reported that linuron, an organochlorine (OC) herbicide that was administered into pregnant female rats caused a decreasing level of testosterone in the male fetuses. The authors suggested that OC most probably induces toxicity directly onto the Leydig cells of the fetus by its ability to inhibit the steroidogenesis, thus disrupting the masculinization of the fetus in the future [70]. Furthermore, testosterone also stimulates epithelium cell proliferation, controlling the synthesis and secretion of growth proteins and influencing AR expression [71].

Moreover, exposure to Methoxychlor causes an abnormal LH hormone secretion that was proven histologically to lead to high polycystic follicles and the absence of corpus luteum in the ovary of female rats [72,73]. A previous study also showed that paternally administered Fenvalerate, a pyrethroid insecticide, on male rats indirectly increased the level of testosterone and estradiol-17ß in the rat progeny [74]. These hormones are important for reproductive development but if excessively produced will give different impacts and damage the reproductive organ itself [75]. Although FNT is recognized as an antiandrogenic agent that causes disruption to the AR, which is abundantly found in the epididymis, prostate gland, and seminal vesicle, the current study did not find any histological changes caused by FNT on these F1 progenies. This might be due to the differences on how the pesticide is exposed to the rats either during paternal or maternal exposures. 

Meanwhile, the present results also did not show any morphological changes on other organs such as the heart, liver, lungs, and spleen of the progenies in FNT-10 and FNT-20 groups when compared with the control group of F1 progeny. However, there was a shrinkage in the glomerulus size and dilation of Bowman’s space observed in both of the FNT groups in the male F1 progenies. A previous study found that female rats injected with sperm having fragmented DNA produced progeny with defects such as organomegaly on the hearts and kidneys as well as tumors on both the lungs and spleen [29]. Kishigami and colleagues [28] concluded that DNA methylation in fragmented sperm is one of the epigenetic modifications that contributes to the organomegaly and tumor formations. Epigenetic changes are necessary to direct normal cellular development and differentiation in developing organisms, however, developmental abnormalities may occur in response to inappropriate epigenetic signaling [76]. Besides, maternal exposure to lufenuron, an insecticide, during organogenesis had caused glomerular shrinkage in the progenies, which might be due to the genotoxic stress and cell cycle arrest of this insecticide [77]. This might explain the possible mechanism involved in the paternal exposure of FNT, which caused glomerulus shrinkage in the male F1 progeny rats. 

## 5. Conclusions

The gradual deterioration of male reproductive quality as a result of environmental toxicity has become a worldwide phenomenon, creating health issues. FNT, a type of OP, can cause significant reproductive impairment, which may be attributable to sperm DNA fragmentation. Some of the F1 progeny’s organs showed defects such as at the testis and kidneys. Furthermore, F1 progeny in the FNT-20 groups also showed some other defects as proven by the anomalously short or absent tail. Further investigations could be done on the effects of FNT in male rats, possibly in terms of the genetic profile through epigenetic studies to determine the exact mechanism causing the impairment.

## Figures and Tables

**Table 1:** Johnsen score.

Score	Stage of Spermatogenesis
1	Tubular sclerosis; absence of seminiferous epithelial cells.
2	Sertoli cells only; no germ cells.
3	Only spermatogonia.
4	Arrest of spermatogenesis at the primary spermatocyte stage; no spermatids.
5	Many spermatocytes; no spermatids.
6	No late spermatids; arrest of spermatogenesis at the spermatid stage.
7	Many early spermatids; no late spermatids.
8	Few late spermatids.
9	Disorganized tubular epithelium with many late spermatids.
10	Full spermatogenesis.

**Table 2:** Sperm characteristics of paternal rats in all experimental groups.

Parameter	Control	FNT-10	FNT-20
Sperm Count (×10^6^)	65.48 ± 1.89	53.00 ± 1.31 ^a^	46.52 ± 1.12 ^a,b^
Sperm Motility (%)	43.59 ± 1.34	20.74 ± 0.67 ^a^	14.10 ± 0.67 ^a,b^
Sperm Viability (%)	60.48 ± 1.20	43.19 ± 1.55 ^a^	35.62 ± 1.19 ^a,b^
Abnormal Sperm Morphology (%)	18.48 ± 1.30	26.10 ± 0.67 ^a^	33.83 ± 0.33 ^a,b^
Sperm DNA Fragmentation (%)	6.90 ± 0.61	12.00 ± 0.52 ^a^	20.91 ± 0.38 ^a,b^

Data are presented as mean ± SEM (one-way ANOVA followed by Tukey post hoc test). Significant difference among groups, ^a^ *p* < 0.05 vs. *p*Control, ^b^ *p* < 0.05 vs. *p*FNT-10.

**Table 3:** Developmental landmarks of F1 progeny in all experimental groups.

Parameter	*p*Control	*p*FNT-10	*p*FNT-20
Anogenital Distance, AGD (mm)			
PND0			
Male	4.13 ± 0.30	3.87 ± 0.30	3.75 ± 0.16
Female	1.88 ± 0.23	1.88 ± 0.23	1.63 ± 0.18
PND12			
Male	14.88 ± 0.30	14.75 ± 0.31	14.63 ± 0.18
Female	8.75 ± 0.37	8.50 ± 0.42	8.38 ± 0.46
PND35			
Male	29.38 ± 0.38	29.25 ± 0.31	28.50 ± 0.63
Female	18.75 ± 0.41	18.88 ± 0.55	18.63 ± 0.42
Number of Nipple and Areola			
PND12			
Male	0.00 ± 0.00	0.00 ± 0.00	0.00 ± 0.00
Female	12.13 ± 0.22	12.00 ± 0.19	11.88 ± 0.13

Data are presented as mean ± SEM (one-way ANOVA).

**Table 4:** Testicular morphometry and spermatogenesis evaluation of F1 progeny in all experimental groups.

Parameter	*p*Control	*p*FNT-10	*p*FNT-20
Leydig Cell Count	208.44 ± 1.46	207.13 ± 1.50	201.94 ± 1.59 ^a^
Seminiferous Tubule Diameter (µm)	250.86 ± 1.27	248.24 ± 1.56	243.59 ± 2.33 ^a^
Seminiferous Tubule Height (µm)	78.67 ± 2.46	75.85 ± 2.28	71.33 ± 1.02
Seminiferous Tubule with Germ Cell Loss (%)	2.72 ± 0.29	2.81 ± 0.23	2.97 ± 0.26
Johnsen Testicular Biopsy Score	9.25 ± 0.25	9.13 ± 0.23	9.00 ± 0.27

Data are presented as mean ± SEM (one-way ANOVA, followed by Tukey post hoc test). Significant difference among groups, ^a^ *p* < 0.05 vs. *p*Control.

**Table 5:** Cardiomyocytes and hepatocytes size, glomerulus diameter, and Bowman’s space as well as the number of alveoli of F1 progeny in all genders of experimental groups.

Parameter	Male			Female		
	*p*Control	*p*FNT-10	*p*FNT-20	*p*Control	*p*FNT-10	*p*FNT-20
Cardiomyocyte Size (×10^3^ µm^2^)	9.93 ± 0.36	10.03 ± 0.17	9.97 ± 0.30	9.54 ± 0.18	9.59 ± 0.11	9.60 ± 0.18
Hepatocyte Size (%)	98.13 ± 0.13	97.50 ± 0.09	95.63 ± 0.11	96.25 ± 0.16	95.63 ± 0.15	95.63 ± 0.11
Glomerulus Diameter (µm)	38.70 ± 1.08	31.49 ± 0.68 ^a^	29.78 ± 0.36 ^a^	34.69 ± 0.24	34.10 ± 0.36	34.03 ± 0.25
Bowman’s Space (µm)	535.41 ± 4.03	551.20 ± 0.85 ^a^	569.98 ± 2.40 ^a,b^	508.41 ± 2.47	502.21 ± 2.11	502.08 ± 1.58
Number of Alveoli (×10^−3^)	14.53 ± 0.31	14.38 ± 0.36	14.31 ± 0.24	13.68 ± 0.12	13.64 ± 0.14	13.65 ± 0.31

Data are presented as mean ± SEM (one-way ANOVA, followed by Tukey post hoc test). Significant difference among groups, ^a^ *p* < 0.05 vs. *p*Control, ^b^ *p* < 0.05 vs. *p*FNT-10.

## Data Availability

Not applicable.

## Supplementary Materials

The following are available online at https://www.mdpi.com/article/10.3390/toxics9070159/s1, Figure S1: Testis, epididymis, prostate gland, seminal vesicle and ductus deferens cross section of rats, stained with H&E (Magnification: 40×), Figure S2: Ovary and uterus cross section of rats, stained with H&E. (Magnification: 40×), Figure S3: Lung and spleen cross section of rats, stained with H&E. (Magnification: 40×), Table S1: Absolute and relative organ weights of F1 progeny in all experimental groups, Table S2: Epididymis, prostate gland, seminal vesicle, ductus deferens and endometrium epithelial height as well as wall thickness endometrium of F1 progeny in all experimental groups.

## Abbreviations

F0	Paternal
F1	First-generation progeny
FNT	Fenitrothion
FNT-10	Group receiving 10 mg/kg Fenitrothion
FNT-20	Group receiving 20 mg/kg Fenitrothion
*p*Control	Progeny of control group
*p*FNT-10	Progeny of paternal receiving 10 mg/kg Fenitrothion
*p*FNT-20	Progeny of paternal receiving 20 mg/kg Fenitrothion
DNA	Deoxyribonucleic acid
OP	Organophosphate
AChE	Acetylcholinesterase
ACh	Acetylcholine
ROS	Reactive oxygen species
HPG	Hypothalamic-pituitary gonadal
KTX	Ketamine and xylazine cocktail
HBSS	Hank’s balanced salt solution
WHO	World Health Organization
IRDG	Industrial reproductive toxicology discussion group
AGD	Anogenital distance
PND	Postnatal day
H&E	Haematoxylin and eosin
MJTBS	Mean Johnsen testicular biopsy score
ANOVA	One-way analysis of variance
SEM	Mean ± standard error of the mean
PUFA	Polyunsaturated fatty acid
FAAH	Fatty acid amide hydrolase
DFI	DNA fragmentation index
BUP	Bupropion hydrochloride
AR	Androgen receptor
3MNP	3-methyl-4-nitrophenol
OC	Organochlorine
LH	Luteinizing hormone
