# Supplementary material for: Paternal Fenitrothion Exposures in Rats Causes Sperm DNA Fragmentation in F0 and Histomorphometric Changes in Selected Organs of F1 Generation
Source: Toxics. 2021 Jul 5;9(7):159. doi: 10.3390/toxics9070159 (PMC8309826; doi:10.3390/toxics9070159)
Supplement: Supplementary file 1 [file toxics-09-00159-s001.zip › toxics-1215454-supplementary.pdf]

toxics

MDPI

Article

Supplementary Materials: Paternal Fenitrothion Exposures in Rats Causes Sperm DNA Fragmentation in F0 and Histomorphometric Changes in Selected Organs of F1 Generation

Nur Afizah Yusoff, Izatus Shima Taib, Siti Balkis Budin and Mahaneem Mohamed

Table S1. Absolute and relative organ weights of F1 progeny in all experimental groups.

Parameter	Male			Female		
	*p*Control	*p*FNT-10	*p*FNT-20	*p*Control	*p*FNT-10	*p*FNT-20
Absolute Liver Weight (g)	11.45 ± 0.44	9.98 ± 0.48	8.25 ± 0.27[a,b]	7.12 ± 0.18	7.11 ± 0.26	6.79 ± 0.14
Relative Liver Weight (%)	3.14 ± 0.10	2.85 ± 0.15	2.47 ± 0.15[a]	2.47 ± 0.06	2.69 ± 0.10	2.65 ± 0.06
Absolute Renal Weight (g)	2.39 ± 0.09	2.20 ± 0.03	2.11 ± 0.12	1.66 ± 0.03	1.56 ± 0.04	1.50 ± 0.05[a]
Relative Renal Weight (%)	0.66 ± 0.02	0.63 ± 0.01	0.63 ± 0.04	0.58 ± 0.01	0.59 ± 0.02	0.58 ± 0.02
Absolute Heart Weight (g)	1.19 ± 0.02	1.10 ± 0.05	1.02 ± 0.05[a]	0.79 ± 0.02	0.75 ± 0.02	0.70 ± 0.02[a]
Relative Heart Weight (%)	0.33 ± 0.01	0.32 ± 0.02	0.30 ± 0.02	0.28 ± 0.01	0.29 ± 0.01	0.27 ± 0.01
Absolute Lung Weight (g)	2.29 ± 0.06	2.10 ± 0.13	1.99 ± 0.08	1.95 ± 0.12	1.74 ± 0.14	1.61 ± 0.07
Relative Lung Weight (%)	0.63 ± 0.02	0.60 ± 0.04	0.59 ± 0.04	0.68 ± 0.04	0.66 ± 0.06	0.63 ± 0.02
Absolute Spleen Weight (g)	0.78 ± 0.05	0.69 ± 0.03	0.62 ± 0.03[a]	0.77 ± 0.04	0.66 ± 0.05	0.57 ± 0.05
Relative Spleen Weight (%)	0.22 ± 0.02	0.20 ± 0.01	0.19 ± 0.02	0.27 ± 0.02	0.25 ± 0.02	0.24 ± 0.02
Absolute Testis Weight (g)	2.16 ± 0.04	2.15 ± 0.09	2.04 ± 0.12	-	-	-
Relative Testis Weight (%)	0.60 ± 0.02	0.61 ± 0.03	0.60 ± 0.03	-	-	-
Absolute Epididymis Weight (g)	0.64 ± 0.04	0.61 ± 0.02	0.61 ± 0.02	-	-	-
Relative Epididymis Weight (%)	0.18 ± 0.01	0.18 ± 0.01	0.18 ± 0.01	-	-	-
Absolute Ductus Deferens Weight (g)	0.18 ± 0.00	0.17 ± 0.01	0.16 ± 0.01	-	-	-
Relative Ductus Deferens Weight (%)	0.05 ± 0.00	0.05 ± 0.00	0.05 ± 0.00	-	-	-
Absolute Seminal Vesicle Weight (g)	0.97 ± 0.10	0.88 ± 0.07	0.72 ± 0.09	-	-	-
Relative Seminal Vesicle Weight (%)	0.27 ± 0.03	0.25 ± 0.02	0.21 ± 0.03	-	-	-
Absolute Prostate Gland Weight (g)	0.79 ± 0.04	0.72 ± 0.02	0.62 ± 0.03[a]	-	-	-
Relative Prostate Gland Weight (%)	0.22 ± 0.01	0.21 ± 0.01	0.19 ± 0.01	-	-	-
Absolute Uterus Weight (g)	-	-	-	0.49 ± 0.03	0.45 ± 0.02	0.42 ± 0.03
Relative Uterus Weight (%)	-	-	-	0.17 ± 0.01	0.17 ± 0.01	0.15 ± 0.02
Absolute Ovary Weight (g)	-	-	-	0.14 ± 0.00	0.12 ± 0.00	0.11 ± 0.01[a]
Relative Ovary Weight (%)	-	-	-	0.05 ± 0.00	0.05 ± 0.00	0.04 ± 0.00[a,b]

Percentage shows organ weight to body weight index. Data are presented as mean ± SEM (One-way ANOVA, followed by Tukey post-hoc test). *significant difference among group, $^{a}p < 0.05$ vs *p*Control, $^{b}p < 0.05$ vs *p*FNT-10.

Table S2. Epididymis, prostate gland, seminal vesicle, ductus deferens and endometrium epithelial height as well as wall thickness endometrium of F1 progeny in all experimental groups.

Parameter	*p*Control	*p*FNT-10	*p*FNT-20
Epididymis Epithelial Height (µm)	16.69 ± 0.92	17.38 ± 0.75	17.41 ± 0.74
Prostate Gland Epithelial Height (µm)	19.94 ± 0.37	19.88 ± 0.30	19.61 ± 0.39
Seminal Vesicle Epithelial Height (µm)	16.89 ± 0.29	16.16 ± 0.63	16.05 ± 0.23
Ductus Deferens Epithelial Height (µm)	25.33 ± 0.58	25.22 ± 0.64	25.17 ± 0.48
Endometrium Epithelial Height (µm)	17.23 ± 0.32	17.04 ± 0.43	17.16 ± 0.46
Endometrium Wall Thickness (µm)	301.84 ± 2.23	300.13 ± 1.93	301.58 ± 2.24

Data are presented as mean ± SEM (One-way ANOVA).

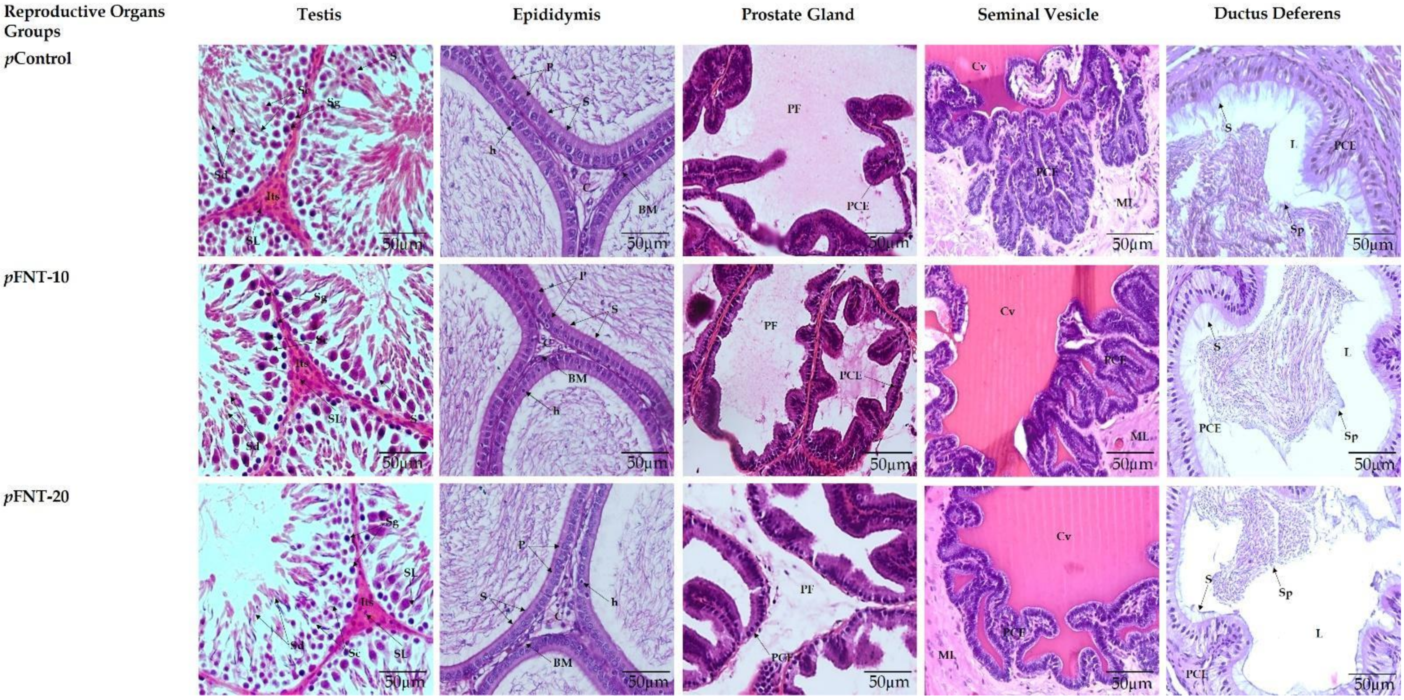

Figure S1. Testis, epididymis, prostate gland, seminal vesicle and ductus deferens cross section of rats, stained with H&E (Magnification: 40x). Normal structure of seminiferous tubules (ST) containing spermatid, Sd (arrow), lumen (L) and germ epithelium layer (GE) in *p*Control and *p*FNT-10 groups. Sd was fully occupied by the seminiferous tubules (ST) of *p*Control and *p*FNT-10 groups. Spermatocyte (Sc), spermatogonia (Sg), spermatid (Sd) and Sertoli cells (S) can be observed clearly in the tubules. Besides, the Leydig cells (SL) (arrow) can be seen in interstitial space (Its) of all experimental rat groups. Epididymis tubule in orderly arrangement and layered by pseudostratified columnar epithelium cells (PCE) with packed mass of spermatozoa (Sp) in all experimental rats. Columnar Principal cell (P) in shape was observed with stereocilia (S) cell. Halo (h) cells were seen scattered between P cells and transparent cytoplasm. Basal membrane (BM) and connective tissue (C) were observed in *p*FNT-10 and *p*FNT-20 groups although no obvious morphological changes in epididymis were seen compared to the pControl group. Epithelial height was normal in all groups. The lumen contains prostatic liquid (PF) surrounded by folded epithelium that later forms glands in *p*Control and *p*FNT-10 groups. Epithelium consists of pseudostratified to columnar epithelium cells (PCE) that are closely lined on the basal membrane (arrow). Epithelium of the prostate appears thinner and less folded with less PF secretion in few areas of *p*FNT-20 group. Mucous layers (ML) are thin in structure, branching out and folded to form uneven anastomosis branches (Mf). Branching pseudostratified glandular epithelium cells (PCE) can be seen in all groups on magnification 40x. The lumen is filled with vesicle fluid (Cv). Overall, no morphological changes were observed in *p*FNT-10 and *p*FNT-20 groups. Normal lumen (L) of ductus deferens is lined with pseudostratified columnar epithelium cells (PCE). Long stereocilia (S) and spermatozoa (Sp) can also be observed in all experimental groups.

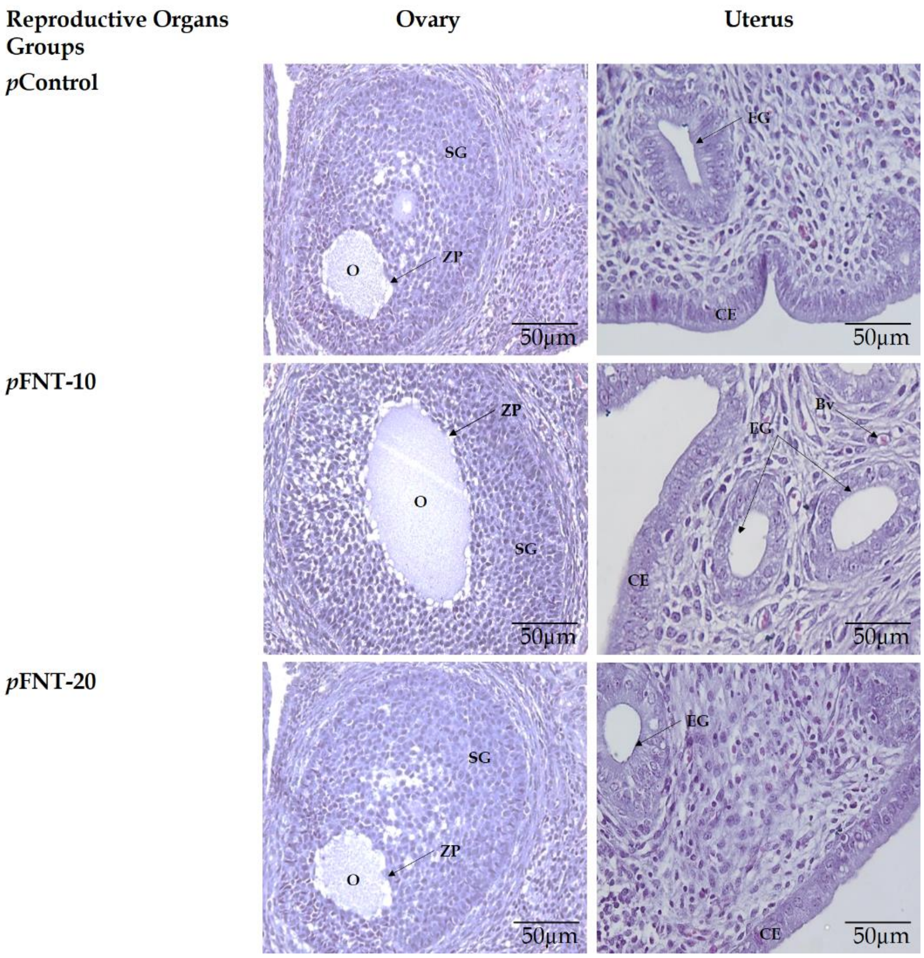

Figure S2. Ovary and uterus cross section of rats, stained with H&E. (Magnification: 40x). Ovary looks normal with the presence of round oocytes (O) with cytoplasm as well as zona pellucida (ZP). Granulosa cells (SG) as polyhedral cells were observed fulfilling and surrounding the O in *p*Control, *p*FNT-10 and *p*FNT-20 groups. Normal endometrial gland (EG) is located outside the endometrium lining that is lined with columnar epithelium (CE), lumen (L), blood vessel (Bv) and endometrium (E) in all experimental groups.

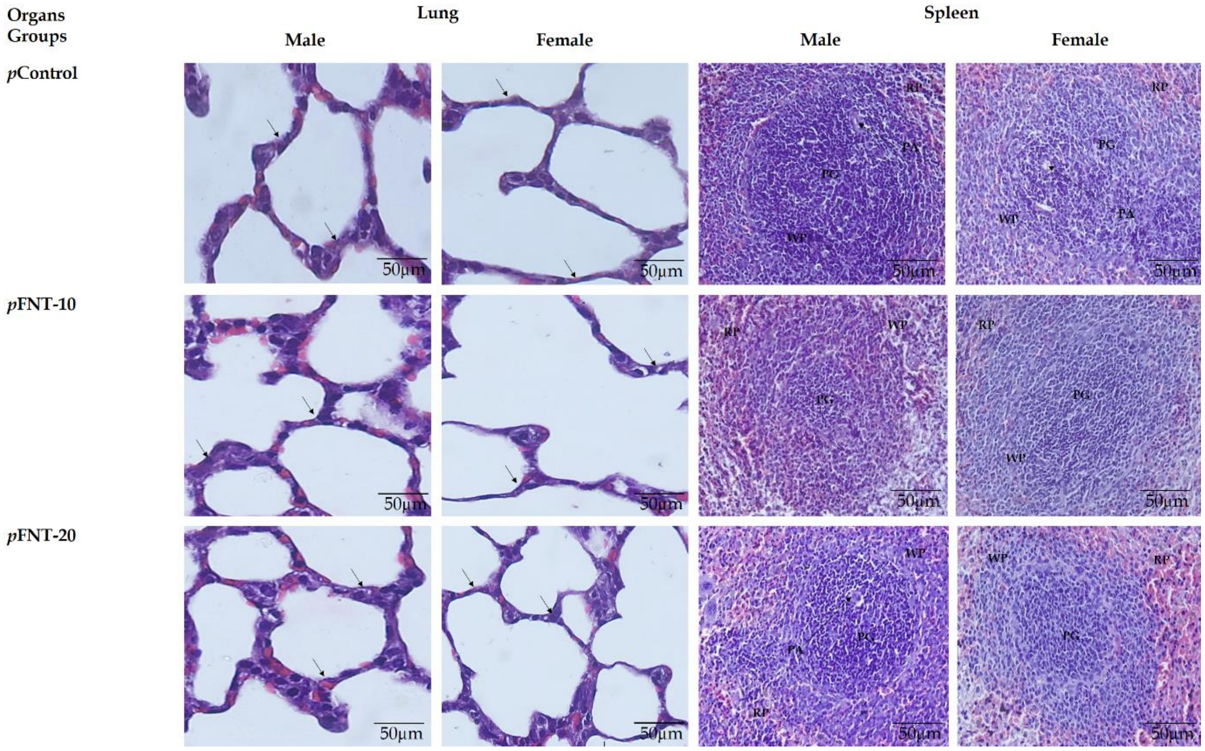

Figure S3. Lung and spleen cross section of rats, stained with H&E. (Magnification: 40x). Normal morphology of spleen with red pulp (RP) due to the presence of erythrocyte in the blood vessel. It surrounds white pulp (WP), which is blue in color in all groups. The arteriole center is located in a few areas of WP. Meanwhile, the germinal center (PG) is located in the middle and surrounded by WP.
